# Integrative genetic map of repetitive DNA in the sole *Solea senegalensis* genome shows a Rex transposon located in a proto-sex chromosome

**DOI:** 10.1038/s41598-019-53673-6

**Published:** 2019-11-20

**Authors:** Emilio García, Ismael Cross, Silvia Portela-Bens, María E. Rodríguez, Aglaya García-Angulo, Belén Molina, Angeles Cuadrado, Thomas Liehr, Laureana Rebordinos

**Affiliations:** 1 Área de Genética, Facultad de Ciencias del Mar y Ambientales, INMAR, Universidad de Cádiz, 11510 Cádiz, Spain; 2 Department of Biomedicine and Biotechnology, University of Alcala, 28871 Alcalá de Henares (Madrid), Spain; 3 Institut für Humangenetik, Universitätsklinikum Jena, 07747 Jena, Germany

**Keywords:** DNA transposable elements, Genomics

## Abstract

Repetitive sequences play an essential role in the structural and functional evolution of the genome, particularly in the sexual chromosomes. The Senegalese sole (*Solea senegalensis*) is a valuable flatfish in aquaculture albeit few studies have addressed the mapping and characterization of repetitive DNA families. Here we analyzed the Simple Sequence Repeats (SSRs) and Transposable elements (TEs) content from fifty-seven BAC clones (spanning 7.9 Mb) of this species, located in chromosomes by multiple fluorescence *in situ* hybridization (m-BAC-FISH) technique. The SSR analysis revealed an average density of 675.1 loci per Mb and a high abundance (59.69%) of dinucleotide coverage was observed, being ‘AC’ the most abundant. An SSR-FISH analysis using eleven probes was also carried out and seven of the 11 probes yielded positive signals. ‘AC’ probes were present as large clusters in almost all chromosomes, supporting the bioinformatic analysis. Regarding TEs, DNA transposons (Class II) were the most abundant. In Class I, LINE elements were the most abundant and the hAT family was the most represented in Class II. Rex/Babar subfamily, observed in two BAC clones mapping to chromosome pair 1, showed the longest match. This chromosome pair has been recently reported as a putative sexual proto-chromosome in this species, highlighting the possible role of the Rex element in the evolution of this chromosome. In the Rex1 phylogenetic tree, the Senegalese sole Rex1 retrotransposon could be associated with one of the four major ancient lineages in fish genomes, in which it is included *O. latipes*.

## Introduction

A large part of the eukaryotic genome is composed of the so-called repetitive DNA, comprising multiple copies of DNA sequences. Repetitive DNA can be divided into two groups: tandem repeats, which includes satellite DNA, and transposable elements (TEs) that are scattered interspersed repetitions^1^. The majority of these repetitive non-coding sequences are usually located in heterochromatic regions. Greater knowledge of these regions and, in general, of the genomes of numerous organisms is now available thanks to high-performance sequencing; this, in recent years, has facilitated great advances in the fields of functional^2^ and comparative genomics^3^. For some time, repetitive sequences were considered to be mainly “junk” DNA^4^, however, with the advances in genomic research, these repetitive sequences are now known to play a more important role in the functional and structural evolution of the genome. Repetitive sequences are implicated in chromosomal rearrangements and are responsible for a substantial proportions of the karyotype variability observed in several groups^5^.

Microsatellites, or simple sequence repeats (SSR), constitute a unique type of genomic sequence repeated in tandem and are repetitive non-coding DNA regions consisting of small motifs of 1 to 6 tandem-repeated nucleotides; they are abundantly distributed throughout all eukaryotic and prokaryotic^6^ genomes. SSRs contribute to the structure of DNA, the organization of chromatin, the regulation of transcription and translation, as well as DNA recombination, and cell cycle dynamics. SSR are present both in the coding and non-coding DNA^6^, although it has been demonstrated that, in eukaryotic organisms, SSRs are mostly in non-coding regions.

TEs are discrete DNA fragments that have the ability to move within a host genome, often creating new copies of themselves during the process. This unique ability of TEs to make copies of themselves seems to be an effective strategy for self-preservation, and this is evident by their presence in all genomes throughout the tree of life^7^. TEs play important roles and can alter or disrupt the expression of genes, promoting population-level variation and rapid adaptation through the expansion of new TE families by generating structural genomic diversity between populations^8,9^. In this sense it has been proposed that the diversity and speciation of teleosts is a reflection of the diversity in the size and structure of their genomes^10,11^.

Depending on the transposition mode (with or without an intermediate RNA) the TEs are divided into Class I and II. Each class comprises different subclasses, superfamilies and families^3^. Among vertebrates, teleosts have the highest number of TE superfamilies^12^ and their abundance seems to be determinant in the size of the genomes of this group^3,13^. Among actinopterygians, teleost fish have the greatest diversity, with more than twenty-five TE superfamilies described in some species^14^. It should be noted that in some species of fish with a small genome size, as pufferfishes and fugu, their genomic reduction is not accompanied by a loss of superfamilies of transposable elements, as is the case in other vertebrates with a reduced genome^13^.

Class I TEs, also called retrotransposons, are characterized by moving through the genomes by means of a copy-paste mechanism, through a reverse transcription of an intermediate RNA. This class is further subdivided into LTR (Long Terminal Repeat) and non-LTR retrotransposons^15^. Based on both structure and phylogenetic data, some differences in reverse transcriptase LTR have been found, and they appear to indicate a close relationship between LTR retrotransposons and some types of retroviruses with more distant non-LTR retrotransposons^3^. The Rex transposable elements are mobile genetic elements belonging to class I^16,17^. Rex elements have been found, in addition to several other TEs, in the genome of some fish species, such as the Nile Tilapia *Oreochromis niloticus*^18^. These elements are generally dispersed and may be related to sexual differentiation within the species. In this sense, an interesting case of a Rex TE type in association with Tc-1 has been detected in the differentiation of the sex chromosomes of *Chionodraco hamatus*, where it was suggestted that transposons in that species may have exerted some influence on the differentiation and structure of sex chromosomes^19^. Class II transposons, or DNA transposons, are divided into three subclasses according to their transposition mechanism: first, “cut-and-paste” transposons; second, inverted terminal repeat sequence (ITRs) transposons (e.g. hATs and Helitrons); and third, self-synthesizing DNA transposons (e.g. Mavericks)^1^.

The large superfamily of hAT transposons, so called from three of its members: the *hobo* element of *Drosophila*, the *Activator* element of *Zea mays* i.e. maize (reported by McClintock) and the *Tam3* element of snapdragon, is very widespread in plants and animals^20,21^. The hAT transposons are also found in the genomes of mammals, and in humans they are the most abundant DNA transposons accounting for 1.55% (195 Mb) of the total genome^22^. The hAT elements have many common features, some of which are subterminal repeats (STRs) at both ends of the TE, inverted terminal repeats (ITRs) and a gene that encodes a 600–800 amino acid transposase which catalyzes the DNA division and integration of the target, with 8 bp in the destination site of duplications (TSD) at each end of the integration site during the transposition^21,23^.

Senegalese sole (*Solea senegalensis*) is considered to be one of the most economically valued fish species in southern Europe. From the beginning of its culture in aquaculture, several problems have appeared, among which the control of reproduction stands out, in particular the infertility present in the individuals grown in captivity^24^. *S. senegalensis* has 21 pairs of chromosomes, and lacks morphologically identificable heteromorphic sex chromosomes^25^ that can be identified by their morphology. The species is male heterogametic having an XX/XY^26^ system and the largest chromosome, originated by the fusion of 2 acrocentric chromosomes^27,28^, has been proposed as a proto-sex chromosome^29^.

Sexual chromosome evolution, from autosomes, is accompained by loss of genes, accumulation of sex-specific alleles and a gain of repetitive DNA sequences^30^. Lack of recombination between heterogametic chromosomes is the mechanism that promotes differentiation between both chromosomes. Co-evolution betweeen TE and recombination has been proposed^31^.

Recently, important advances in genomic tools and resources have been documented in some of the main cultured flatfish species^32^. These resources include whole genome sequencing, genetics maps, QTL studies and mapped SNPs. However, with the exception of one genetic map^26^ and some cytogenomics maps based in BAC-FISH^27–29,33^ few studies exist dealing with structural genomics and sequencing in the Senegalese sole and only the abundance and type of microsatellites present in its transcriptome have been described^34^.

The objective of this work is to provide new information about the distribution of microsatellite motifs (dinucleotides, trinucleotides, tetranucleotides, pentanucleotides and hexanucleotides) and main motifs of TE in the genome of *S. senegalensis* paying special attention to the largest metacentric chromosomes proposed as proto-sex chromosome. With this information we discuss the aspects of the organization of TE and microsatellites in the sole genome. In particular, we discuss the role and abundance of Rex transposons in the major chromosomal pair of the *S. senegalensis* genome as a possible proto-sex chromosome.

## Methods

### PCR screening from *S. senegalensis* genomic library and BAC clone sequencing

Thirty-two BAC clones from a library of the *S. senegalensis* genome previously constructed and described were screened^29,33^. The BAC library is comprised of 29,184 clones distributed in 384-well plates (76 plates in total). BAC clones were identified and isolated using a 4D-PCR method. Briefly, plates were organized in 4 pools and used as template DNA. Primers for several gene and sequences were used to carry out the screening of the pools in a hierarchical way until location of the BAC in an specific coordinates of a specific plate of the library. Each BAC clone was named using the library plate number, and columns and rows coordinates. Clones were used in m-FISH experiments and in repetitive sequence analysis. BAC clones were sequenced as described in Garcia-Cegarra *et al*.^33^. Briefly, DNA from the *S. senegalensis* BAC genome library was isolated and purified using the Large-Construct Kit (Qiagen, Hilden, Germany), and then digested with *Hae* II and *Rsa* I enzymes (20 U). A total of 454 sequencings were performed according to supplier’s recommendations.

BAC sequences from another twenty-five BAC clones previously described^28,29,33^ were used for the repetitive sequences study and integrated mapping analysis. BACs with several chromosome locations were counted as many times as they were localized. Overall, sixty-four BAC clone sequences have been analyzed in this work, integrating information about their chromosome localization, number and distribution of SSRs and TEs (Accession Numbers AC278047-AC278120).

The experimental procedures were in accordance with the recommendation of the University of Cádiz (Spain) for the use of laboratory animals (https://bit.ly/2tPVbhY) and the Guidelines of the European Union Council (86/609/EU). The experiment was authorised by the Ethics Committee of University of Cadiz (Spain).

### FISH analysis

#### Chromosome Preparations

Chromosome preparations were made according to Cross *et al*.^35^. Briefly, 2–3 day-old *S. senegalensis* larvae were pretreated with 0.02% colchicine for 3 h. Then they were subjected to hypotonic shock with KCl (0.4%) and finally fixed in a freshly-prepared solution of absolute ethanol:acetic acid (3: 1). Larvae were homogenized in Carnoy, and the preparations were then dropped onto wet slides and placed on a hot plate with damp paper to create the necessary moisture for a good spread of the chromosomes^29^.

#### mBAC-FISH

BAC clones labeling was carried out with a first amplification by DOP-PCR, followed by a conventional PCR for labeling, as described previously in Garcia Angulo *et al*.^28^. Three different fluorochromes were used: Texas red (Thermo Fisher Scientific, USA), fluorescein-isothiocyanate (FITC) (Enzo, USA), and diethyl-aminocoumarin (DEAC) (Vysis, USA). The chromosomes were pretreated with pepsin and fixed in formaldehyde. Finally, the chromosome preparation was dehydrated with ethanol series and air-dried before hybridization. Hybridization was done according to Portela-Bens *et al*.^29^.

#### SSRs-FISH

Chromosome preparations were made according to Cuadrado *et al*.^36^. A total of 10 different mono-, di-, tri-, and tetranucleotide microsatellite motifs were physically mapped using synthetic oligonucleotides labeled with biotin at both ends (Roche Applied Science) as probes: (A)_20_, (C)_20_, (AC)_10_, (AG)_10_, (ACG)_5_, (AAT)_5_, (GCA)5, (AAC)_5_, (GACA)_4_, (GATA)_4_. Chromosomes and probe denaturation and *in situ* hybridization were performed as described by Cuadrado *et al*.^36^. In brief, the hybridization mixture was prepared by adding 50% de-ionized formamide, 10% dextran sulfate, 2× SSC, 0.1% sodium dodecyl sulfate (SDS), and 2 ppm of the microsatellite probes. For post-hybridization washing, slides were immersed in 4× SSC/0.2% Tween-20 for 10 min at room temperature (RT). Biotin was detected by incubating the slides in streptavidin-Cy3 (Sigma) in 5% (w/v) BSA for 1 h at 37 °C. Slides were rinsed for 10 min in 4× SSC/0.2% Tween- 20 at RT and then stained with DAPI (4′,6-diamidino-2-phenylindole). They were mounted in Vectashield antifading medium (Serva) and examined with a Zeiss Axiophot microscope.

#### Bioinformatic analysis

SSR and TE analysis. After determining the chromosome location of BAC clones, using the FISH technique, the genomic sequences obtained from those clones (taking into account several multi-loci situations) were loaded into a local pool. A configuration file was used together with the perl script MISA (Microsatellite identification tool)^37^. DNA sequences were then searched for both perfect and compound microsatellites, with a basic motif of 2–8 bp. Only 1 to 6 motifs were considered, and the minimum repeat unit was defined as 10 for mononucleotide, 6 for dinucleotide repeats, and 5 for tri-, tetra-, penta- and hexa-nucleotides. The maximum number of bases interposed between two SSRs in a compound microsatellite was set at 100. A homology-based approach using the Repbase (release 23.07) database; RepeatMasker^38^ was also applied. Analysis of TEs distribution in the Senegalese sole genome was made possible by using both the information of BAC clone position obtained from the FISH technique, and the coordinates of the TE elements from the RepeatMasker software. Statistical analysis to determine frequency and distribution by chromosome of both TE and SSR elements was done using SPSS software (v17.0).

#### Phylogenetic analysis

In order to generate the phylogenetic tree for the Rex retrotransposon, fish Rex1 sequences from Repbase (Giri repbase - https://www.girinst.org/) were downloaded. In addition, the BLASTn algorithm^39^ was used in the Ensembl database (https://www.ensembl.org) to find homologies with sequences matching the *S. senegalensis* Rex1 element, and the matched sequences were also used. One hundred and twenty five fish sequences were included in the phylogenetic tree. All sequences were then aligned in MAFFT software^40^ using an iterative method. To eliminate poorly-aligned positions and divergent regions of DNA, the Gblocks server was used, and different options for a less stringent selection (allowing smaller final blocks, allowing gap positions within the final blocks, and allowing less-strict flanking positions) were applied to the analysis. Then the SMS program (Smart Model Selection) was applied to determine the best-fit phylogenetic model^41^ and, finally, the PhyML 3.0 software^42^ was used to run the model. The resulting best-fit model predicted was GTR + G + I. The proportion of invariable sites was 0.012, the number of substitution rate categories was 4, and the Gamma shape parameter estimated was 1.389. The statistic used for model selection was the Akaike information criterion (AIC), the value of which was 235939.11 and the -LnL was -117712.55986. Branch support was tested by the fast likelihood-based method using aLRT SH-like^43^ Tree edition was carried out using MEGA version 7^44^.

### Compliance with ethical standards

The experimental procedures are according to the recommendation of the University of Cádiz (Spain) for the use of laboratory animals and the Guidelines of the European Union Council (86/609/EU). The experiment was authorised by the Ethics Committee of University of Cadiz (Spain).

## Results

### BAC-FISH mapping in *S. senegalensis*

**Figure 1:**
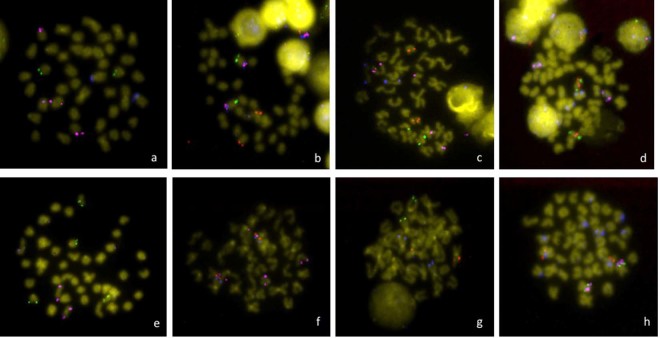
Metaphase plates with the locations of the BACs used in this study: (**a**) 36-E3 (*blue*), 31-A1 (*green*), 31-N1 (*pink*); (**b**) 36H3 (*pink*), 36-J2 (*blue*), 46-C5 (*red*), 12-N5 (*green*); (**c)** 4F-12 (*green*), 4-E10 (*red*), 36-K1 (*pink*), 31-N1 (*blue*); (**d)** 13-F2 (*red*), 35-D17 *(green*), 31-N1 (*pink*), 31-A2 (*blue*); (**e**) 36-E3 (*green*), 31A1 (*blue*), 31-N1 (*pink*); (**f**) 13-G1 (*green*), 36-H2 (*red*), 36-M2 (*pink*), 36-J2 (*blue*); (**g**) 31A2 (*green*), 36-I3 (*red*), 36-H3 (*blue*); (**h**) 52-C17 (*red*), 48-K7 (*blue*), 36-I3 (*green*), 46-C5 (*pink*). Underlined BAC clones indicate the probes localized in this study. Other clones were used to map the relative position of all of the sequences to build the integrative map.

**Figure 2:**
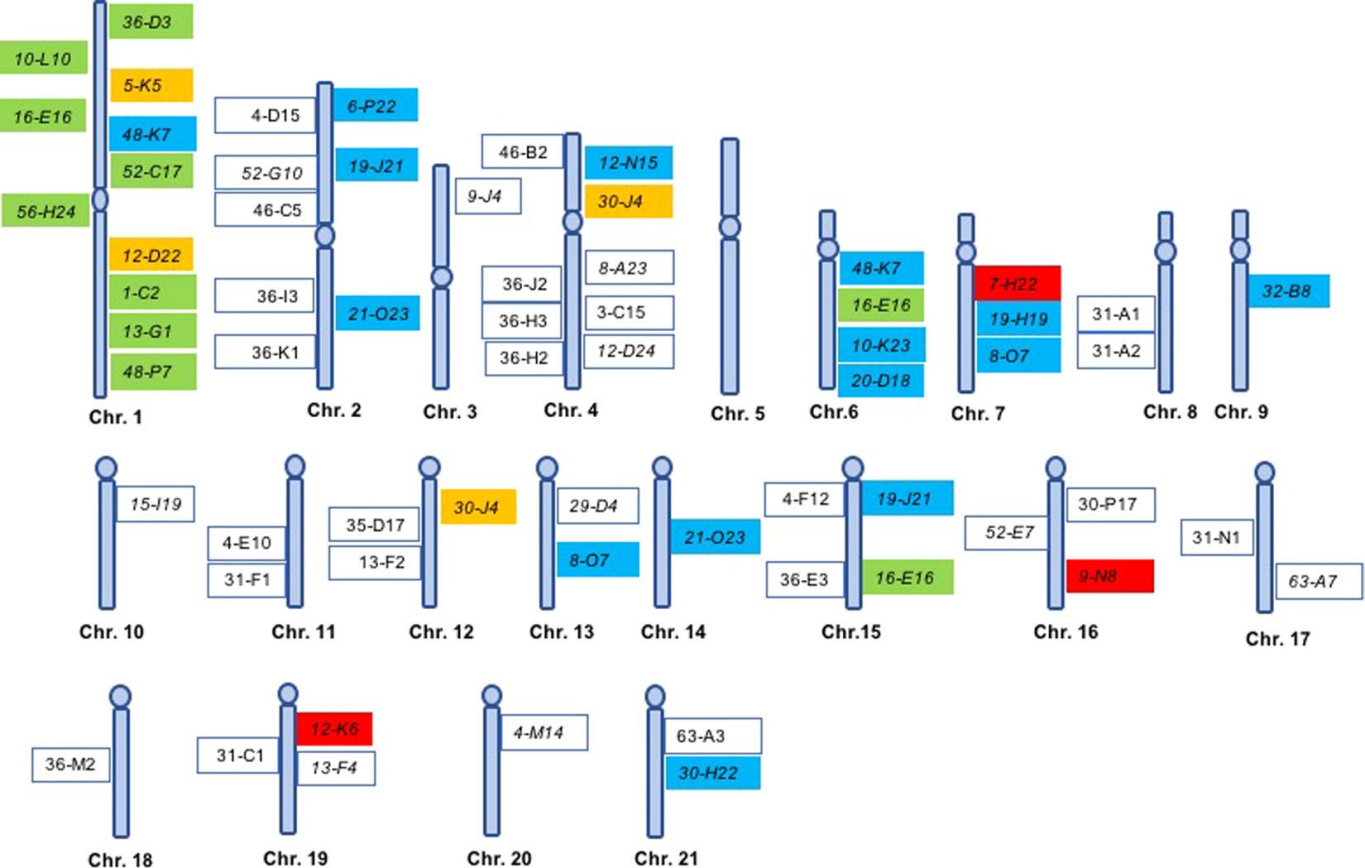
Cytogenetic maps of *S. senegalensis*. Cytogenetic results are shown in boxes within the chromosome diagram; the green boxes indicate the results obtained by Garcia-Angulo *et al*. (2018), blue boxes those by Portela-Bens *et al*. (2017), orange boxes those by García-Cegarra *et al*. (2013), red boxes those by Merlo *et al*., 2017, and white boxes those by this study.

A multi BAC-FISH mapping was performed in *S. senegalensis* chromosomes using 32 BAC clones (Fig. 1) and integrating previously published data^27–29,32^. Results are summarized in Fig. 2 and Supplementary Table S1.

Multiple hybridization analysis showed four clones producing a single signal and not co-localizing with other clones. Specifically, BAC9-J4 presented a signal in the small metacentric chromosome pair 3. The other three BACs appear in acrocentric chromosomal pairs: BAC15-I19 in pair 10; BAC36-M2 in chromosomal pair 18; and BAC4-M14 gives a signal in the chromosomal pair 20.

The hybridization signals of 5 new BACs were observed in the median metacentric chromosome pair number 2. In one arm appear the five BACs 4D-15, 52-G10 and 4C-5 that co-localize with those previously detected by Portela Bens *et al*.^29^. (2017), 6-P22 and 19-J21, the latter also signaling in the acrocentric pair 15. In the other arm, the BACs 36-I3 and 36-K1 signal; these co-localize with the BAC 21-O23 that also signals in the acrocentric pair 14 as a single signal, as described in Portela-Bens *et al*.^29^.

In the submetacentric pair 4, the hybridizations give signals for 7 new BACs. The BAC46-B2, in the short arm in telomeric position, co-locates in the same region as the previously described BACs 12-N15^29^ and 30-J4^32^. Six new BACs were located in the long arm: BAC36-J2, BAC36-H3, BAC36-H2, BAC 8-A23, BAC3-C15 and BAC12-D24. In the subtelocentric chromosome pair 8, two signals co-located: BACs 31-A1 and 31-A2.

In six acrocentric chromosomal pairs, signals from new BACs clones were detected co-localizing with others already described above: in pair 12 BACs 35-D17 and 13-F2 co-localize with the BAC30-J4 located in an almost centromeric position, also detected on chromosome 4^32^. In chromosome pair 13 we detected the BAC29-D4 signal, also located in a more centromeric situation, which co-located with BAC8–07 that is co-localized in the long arm of the subtelocentric chromosome 7^29^. In pair 15 we find 2 signals that correspond to the BACs 4-F-12 and 36-E3 and co-localize with the BACs 19-J21^29^ and 16-E36; the latter co-localizes in the large metacentric chromosome 1 and in the long arm of the subtelocentric chromosome 6^28^. In the chromosomal pair 16 we detect the signals corresponding to the BACs 52-E7 and 30-P17 that co-locate with BAC9-N8^27^. In chromosomal pair 19 we detected the signals of BACs 31-C1 and 13-F4 that co-localize with BAC12-K6;^27^ and finally in pair 21 we find the signal of BAC 63-A3 co-locating with the signal of BAC30-H22^29^.

### FISH mapping of SSRs in *S. senegalensis*

**Figure 3:**
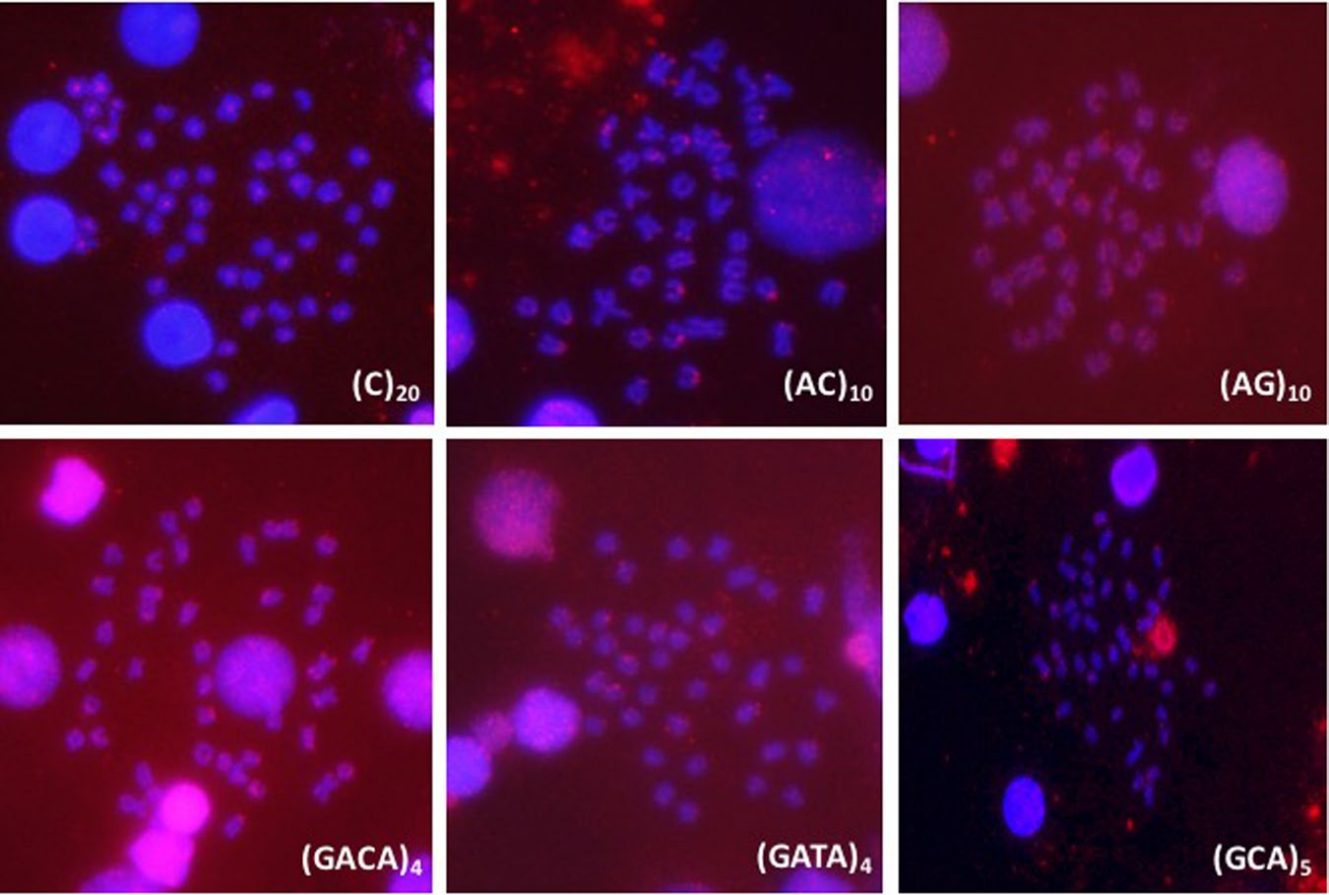
FISH mapping of microsatellite motifs in *S. senegalensis* chromosomes: (C)_20_, (AC)_10_, (AG)_10_, (GACA)_4_, (GATA)_4_, and (GCA)_5_.

To study the distribution of SSR sequences in the *S. senegalensis* genome, 2 mono-, 2 di-, 4 tri- and 2 tetra-nucleotide probes were used (Table 1). Seven out of the 11 probes yielded positive signals: (A)_20_, (C)_20_, (AC)_10_, (AG)_10_, (GCA)_5_, (GACA)_4_, (GATA)_4_ (Fig. 3). (AC)_10_ probe presented the largest and most intense signals. This SSR was found in subtelomeric position both in larger (metacentric and submetacentric) and acrocentric chromosomes. This distribution was similar to the location of the (GACA)_4_ probe, and in a smaller quantity, the GATA repeats. The (AG)_10_ probe displayed a dispersed pattern of FISH signals on chromosomes, showing a greater concentration in subtelomeric positions. Mononucleotide probes (A)_20_ and (C)_20_ were found scattered among several chromosomes: the A probe showed weak signals although with a somewhat greater concentration in subtelomeric positions; the C probe presented clusters in centromeric positions of almost all chromosomes.

**Table 1:** Number of FISH signals and localization of BAC clones onto *S. senegalensis* chromosomes.

SSR probe	Localization of signals	
	Chromosome type	Position within chromosome
A_20_	MT, SMT, STL, A	STL
C_20_	MT, SMT, STL, A	C
AC_10_	MT, SMT, A	STL
AG_10_	M, SMT, STL, A	Dispersed
GACA_4_	MT, SMT, A	STL
GATA_4_	MT, SMT, A	STL
GCA_5_	SMT, A	STL
AAT_5_	−	−

*Cromosome type*.

MT: metacentric chromosome.

SMT: sub-metacentric.

STC: sub-telocentric.

A: acrocentric.

*Position within chromosome*.

STL: subtelocentric.

C: centromeric.

—: absence of FISH signal.

### NGS analysis of SSRs and TEs in the *S. senegalensis* BAC clones

To study the number, distribution and abundance of microsatellites in *S. senegalensis*, fifty-seven BAC clones from a genome library were analyzed with MISA software. Twenty-three out of them had been sequenced previously^27–29^ (Table S1). The 57 clones comprise 6.9 Mb. As described in BAC-FISH results, some BACs were localized in two or more chromosomes, so these were included as many times as they appear in the *S. senegalensis* chromosomes. Taking this into account, the total number of BACs used in the SSR analysis was 64, and the total sequence length analyzed was 7.9 Mb. The number of SSR loci observed was 5330, comprising 1.27% of the genome analyzed, and presenting a total of 53505 repeat units. The average number of loci per Mb was calculated as the total number of identified loci (5330) in relation to the BAC sequences length analyzed (7.9 Mb) and normalized by Mb. In average, 675.1 SSR loci per Mb were found in the *S. senegalensis* genome. The coverage calculated as the quantity of sequences of SSR (bp) in relation to the BAC sequences length analyzed (7.9 Mb), and again normalized by Mb, was 12716.63 bp. Attending to the motif length of microsatellite DNA, the di-nucleotide motif showed the largest number of identified SSRs, with almost thirty thousand repeats (29968) in the Senegalese sole BAC clones, followed by mononucleotide repeats (16246). The mean number of repeats loci was higher for the mononucleotide motif than for the dinucleotide motif (11. 63 and 10.33 respectively). The analysis also showed a high level of dinucleotide coverage (measured as nucleotides per Mb sequenced), with an abundance of 59.69%. The mononucleotide and trinucleotide abundance presented lower values (16.18 and 16.32%) than dinucleotides (59.69%) (Table 2). When microsatellite abundance per motif length class is studied, it can be seen that the mononucleotide “A” is rather more abundant than “C” (81.3% vs 18.7%). In the dinucleotide class, “AC” was the most abundant in the genome analyzed (67.7%). The most abundant trinucleotide motifs were “AAT” and “AGC” (28.4 and 21.8% respectively) (Table 3).

**Table 2:** Number and length of microsatellite loci per motif size, microsatellite coverage and relative abundance per motif in *S. senegalensis*.

Motif length class	Number of repeats (NR)	Total number of identified SSRs (NL)	MNRL	Nucleotides per Mb sequenced	Relative motif abundance (%)
Mononucleotides	16249	1397	11,63	2057,99	16,18
Dinucleotides	29968	2900	10,33	7591,10	59,69
Trinucleotides	5463	790	6,92	2075,72	16,32
Tetranucleotides	1320	175	7,54	668,73	5,26
Pentanucleotides	479	63	7,60	303,33	2,39
Hexanucleotides	26	5	5,20	19,76	0,16
TOTAL	53505,00	5330,00		12716,63	100,00

**Table 3:** Microsatellite abundance (%) per motif length and sequence in the *S. senegalensis* genome.

Motif length class	Motif sequence	Relative abundance (%)
Mononucleotides	A	81,32
	C	18,68
Dinucleotides	AC	67,69
	AG	14,21
	AT	17,76
	CG	0,34
Trinucleotides	AAC	10,00
	AAG	8,48
	AAT	28,35
	ACC	3,29
	ACG	0,51
	ACT	1,01
	AGC	21,77
	AGG	15,32
	ATC	9,87
	CCG	1,39

**Figure 4:**
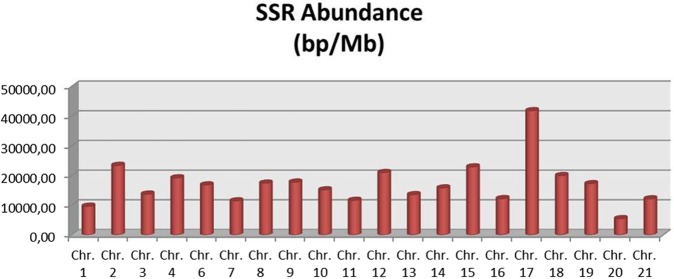
Abundance of SSRs in *S. senegalensis* chromosomes measured as bp of SSR per Mb of genome sequenced.

After positioning BACs on chromosomes by means of FISH, the location and genome abundance of SSRs (measured as bp of SSR per Mb) could be studied (Fig. 4). Chromosome 17 showed the highest SSR coverage, with more than 41000 bp of SSR per Mb. Chromosomes 1 and 20 showed the lowest SSR coverage (9644 and 5457 bp per Mb). When number of loci was measured, similar results were found: chromosomes 1 and 20 show the lowest values and chromosome 17 the highest (Suppl. File 2).

**Figure 5:**
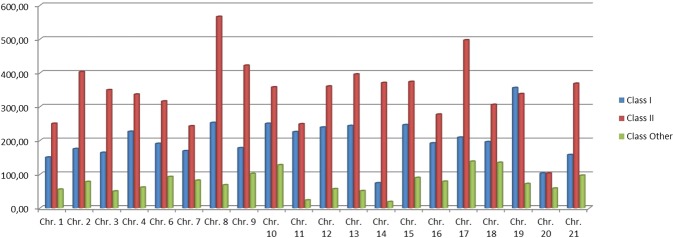
Abundance of TE elements in *S. senegalensis* measured as number of loci per Mb.

All BAC sequences, with information about their chromosome position, were also analyzed using Repeat Masker software. After removing simple repeats and artifacts, 4685 BAC clone positions matching with known Repbase TE elements were obtained. Results were organized by: Class I (retrotransposons); Class II (DNA transposons); and Other repeat elements (Table 4). As it can be observed in Table 4, Class I transposons showed 1549 elements in the genome sampled (BACs sequenced) which represents 33.04% of the TEs in the genome analyzed. From this Class I, 717 elements were found as LINES. Within LINES elements, we found another 14 families, the most abundant being, in numbers of elements (660 out of 717 = 92%) the following: L2 (364), RTE-BovB (112), Rex (88), L1 (63) and Penelope (33). When LINES elements were filtered by length (higher than 1 kb), only Rex and L2 families (5 loci) showed matching repeats (L2:1199–2113 bp and Rex: 2551 bp). When filtering for matches longer than 500 bp, again only these two families were found. The DNA transposons (Class II) were the most abundant with 54.9% of the TEs found, with the hAT family being the element with the greatest presence in the *S. senegalensis* genome: 900 elements and an abundance of 19%. Other repeated elements such as rRNA, tRNA and scRNA show an abundance of 12.06%. Taking into account the genome distribution of TE elements by chromosomes, results showed a heterogeneous distribution (Fig. 5). Chromosomes 8 and 17 showed the greatest abundance with more than 450 loci per Mb. The Class I: Class II ratio was 1.86 on average for all chromosomes, with an extreme value (ratio: 5) in chromosome 14 because of the very low Class I TE value.

**Table 4:** Number and relative abundance of TE elements in the BAC sequences of *S. senegalensis*.

Repeat DNA	Number of elements	Abundance (%)
Class I (retrotransposon)		
LINES	717	15,30
LTRs	364	7,77
SINE	467	9,97
Other (Retroposon)	1	0,02
Total	1549	33,06
Class II (DNA transposons)		
hATs	900	19,21
Tc/Mariner	320	6,83
Other transposon	1193	25,46
Helitron	159	3,39
Total	2572	54,90
Other repeated elements		
Satellite	83	1,77
rRNA	33	0,70
tRNA	20	0,43
Satellite/acro	5	0,11
scRNA	1	0,02
Low_complexity	406	8,67
unknown	17	0,36
Total	565	12,06

**Figure 6:**
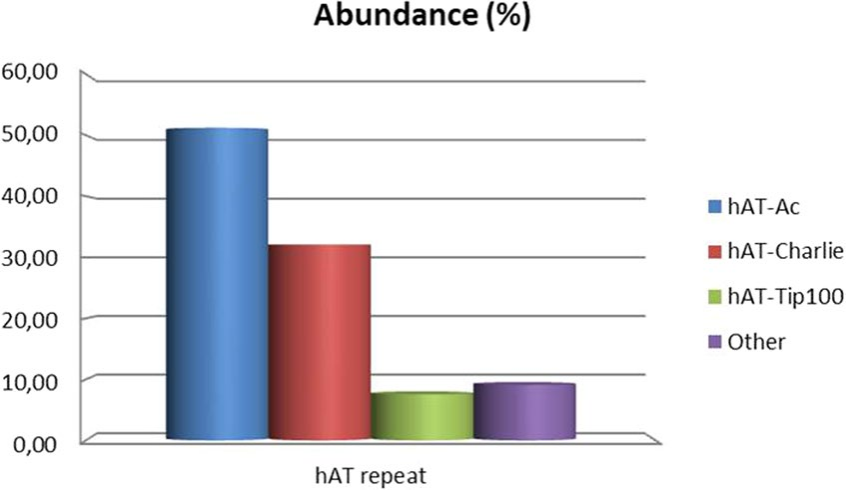
Abundance of Ac, Charlie, Tip100 and others hAT elements measured as percentage of each elements relative to the total number of hAT family elements found in the BAC clone sequences from *S. senegalensis*.

Within TE elements found in the *S. senegalensis* genome analyzed (BAC sequences), hAT elements were the most abundant (900 elements, Table 4). Within hAT, some elements as Charlie, Ac or TIP100 were the most frequent (818 out of 900 elements: > 90%). The Fig. 6 represents the percentage of these elements out of total hAT elements. As it can be observed, the hAT elements from Class II found in the genome showed that more than half of those elements analyzed (51.22%; 461 elements) were hAT-Ac, followed by hAT-Charlie (32.11%; 289 elements), hAT- Tip100 (7.56%; 68 elements) and other repeats of minority elements (9.11% cumulative).

**Figure 7:**
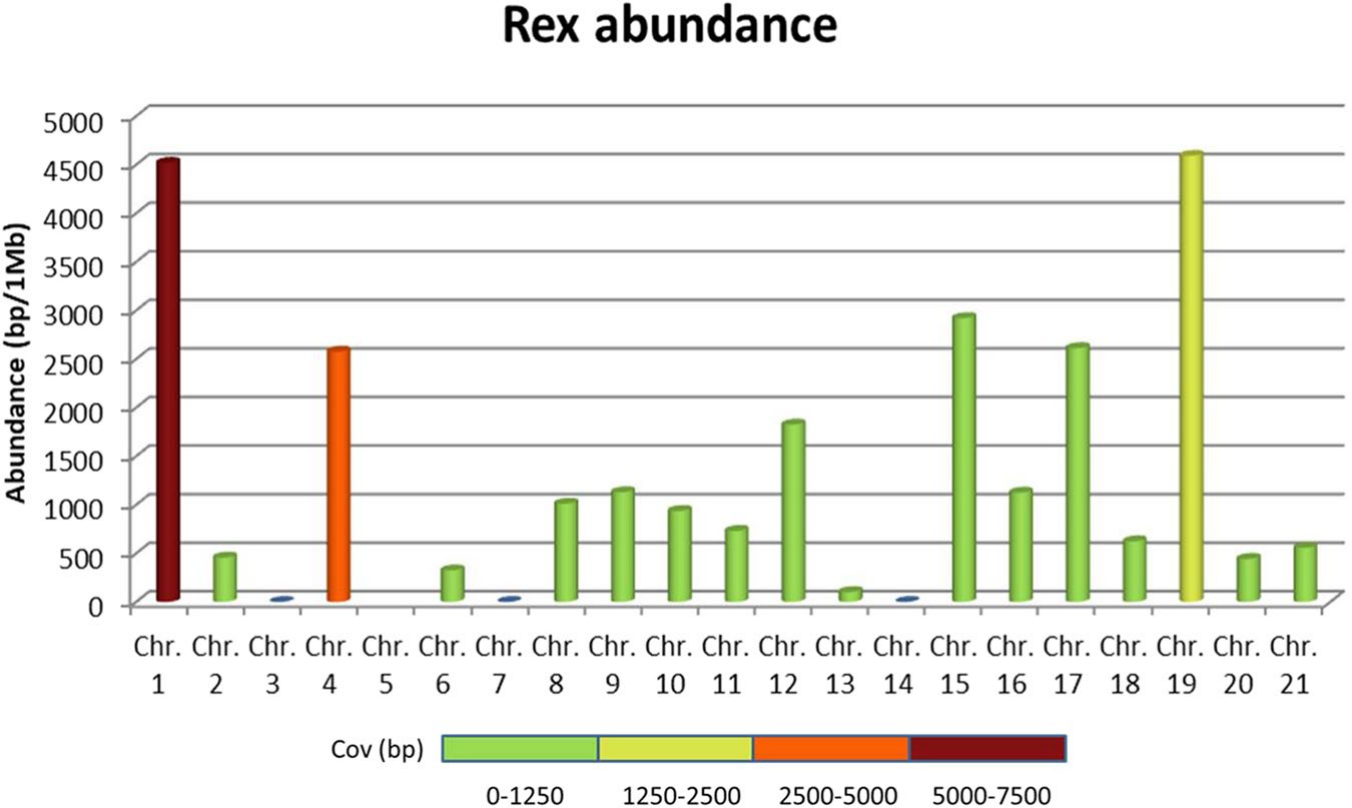
Rex element abundance per chromosome in Senegalese sole measured as coverage (bp) per Mb of total BAC clone sequenced. Colour scale bar shows the coverage of Rex element in bp per chromosome.

After BAC clone sequence analysis, the distribution and abundance of TE elements in the *S. senegalensis* genome was assessed. Within LINES elements, five of them matched regions longer than 1 kb, with the Rex/Babar subfamily showing the longest one (match length 2551 bp). This Rex family was observed in two BACs localized in chromosome 1 (10-L10 and 5-K5). According to recent literature this chromosome could be a proto-sex chromosome. In this sense, we measured the coverage of Rex elements per chromosome, finding the highest value (7427) in chromosome 1, followed by chromosome 4 (3277) and chromosome 19 (1277). In addition, short sequences from BACs of different chromosomes showed similarities with Rex transposon, having a wide distribution across the genome (Fig. 7).

**Figure 8:**
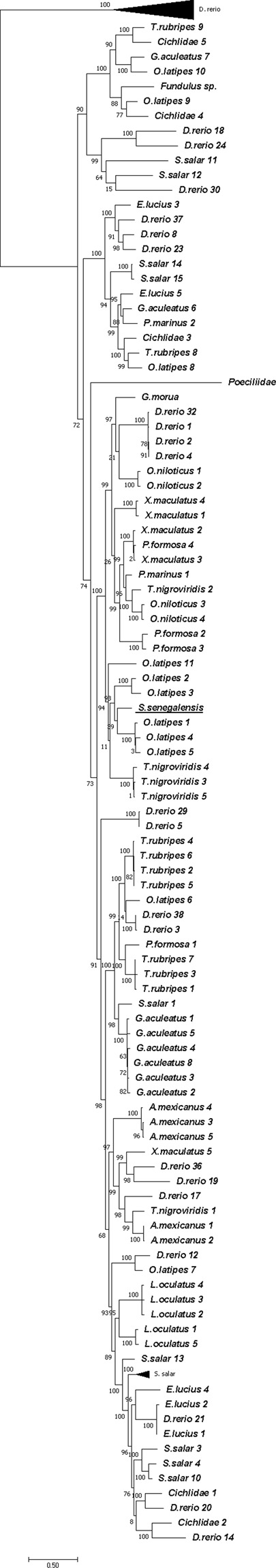
Phylogenetic tree (ML) constructed from bio neighbor-joining, as a starting tree, for the *Rex* retrotransposon found in chromosome 1.

The Rex/Babar sequence, from BACs localized in chromosome 1, was then used against other teleost genomes, as a query in a BLAST search of the Ensemble database. The matches obtained were then extracted as FASTA files and a phylogenetic tree was made (Fig. 8). The tree showed a group with two robust branches: one containing *S. senegalensis*, *Oryzias latipes* and *Tetraodon nigroviridis*, and other containing several sequences from *Danio rerio*, *Salmo salar*, *Gasterosteus aculeatus*, *Takifugu rubripes* and *Lepisosteus oculatus*. In the former, Senegalese sole is present in an internal branch comprising different sequences from *O. latipes*. *T. nigroviridis* shares a branch (low support) with a sequence of *O. latipes*. In a third group, several species such as *O. niloticus*, *Gadus morua* and *Poecilia formosa* can be observed.

## Discussion

In the present work, we studied repeated DNA in *S. senegalensis* using cytogenetic techniques and BAC sequencing. Although the fraction of the Senegalese sole genome studied in the present work is approximately 1.1% of the estimated total size, it can be considered a representative sample to detect the repetitive elements present in the genome of this species^45^. In another species, the pea (*Pisum sativum*), it has been found that a low-pass sequencing of its genome is sufficient to capture the repetitive sequences present in its genome with at least 1000 copies;^46^ and the potential of bioinformatic analysis of low-depth sequencing data for investigation of repeats has been further demonstrated in several other studies^47,48^. Consequently, the analysis of BAC sequences, together with the knowledge of their location by BAC-FISH and supported by the results of SSR-FISH, has enabled us to quantify for the first time the number and distribution of the repetitive elements of the genome of *S. senegalensis*.

In previous studies an integrated genetic map was constructed in the Senegalese sole;^27,29^ this map comprises the sequence and localization of more than 50 BACs. In this study, using the mFISH technique, we determined the chromosome location of 32 new BAC clones and their genome sequences. Using this approach, the main advantage is that it allows us to study the repetitive distribution elements, after BAC hybridization, in various Senegalese sole chromosomes. The results obtained in the present work indicate a frequency of SSRs similar to the data of the transcriptome published^34^. In the genome studied here, dinucleotides were the most abundant motifs (at around 60%), followed by trinucleotides and mononucleotides (both at 16%); in the transcriptome the most abundant microsatellite were dinucleotides, followed by trinucleotides and tetranucleotides in decreasing abundance. The most common SSR motifs in the *S. senegalensis* transcriptome were AC and GT for dinucleotides (at 74.6%) and in the present study they are slightly lower (at 67%). The most abundant trinucleotides in the transcriptome are AGG and CCT (at 21.5%): this finding differs from that detected by BACs (AAT and CCG, at 28.3%)^34^.

In this study, the source of SSRs are BAC clone sequences that have been located throughout the genome of the sole. A total of 5330 microsatellites were identified based on BAC sequences and comprising 1.27% of the genome analyzed, a value similar to that found in the genome of the fugu puffer fish *T. rubripes* (1.29%)^49^ and slightly less than half that in the green puffer fish *T. nigroviridis*, where SSRs account for 3.21% of the genome^50^. These data are consistent with the long-standing assumption that microsatellites are present in all the vertebrate and invertebrate species so far studied. The abundance of microsatellites in sole is similar to that found in humans (>1.5%)^49^ and a little lower than that found in mouse (2%)^51^ and snake (2.8%)^52^.

Although it is widely assumed that the abundance of microsatellites rises with the genome size, many exemptions have been recorded in animals and plants^53,54^. The microsatellite frequency described in this work (on average, 675 loci per Mb) is similar to that obtained in *Drosophila*, with a genome three times smaller (180 GB), in human with a genome five times larger (3000 GB), and in mouse^51^. The relative abundance of length classes of microsatellite motif exhibits a remarkable inter-species variation but dinucleotides and mononucleotides are the predominant in the majority of cases^55,56^. In *S. senegalensis* the dinucleotide motifs are the most abundant, in a proportion of 59.69%. Next, with lower but similar values, we found the mononucleotides and the trinucleotides (16.18 and 16.32% respectively).

The most abundant dinucleotide motifs are AC and GT, a finding similar to that described for the swamp eel genome^57^ and that of human^58^. The AC and GT motifs have been reported as the most frequent SSRs in the intergenic and intron regions of vertebrates^6^ and are 2.3 times more frequent than (AT) n, the second most general type of dinucleotide^6^. The more notable repeats in trinucleotides are AAT, AGC and AGG, and the relative abundance of the AAT motif is the most notable; this also occurs in the swamp eel genome^57^. It shows a predominance of A-rich repeats during the evolution of the genome in teleosts. The extent of the repeats is probably affected by their secondary structures and the influence on DNA replication;^58^ or it could reflect a genetic adaptation to the aquatic environment during speciation of fish. Trinucleotide, tetranucleotide, penta- and hexanucleotide microsatellites are much less frequent than dinucleotides and are usually present 1 to 5 times less frequently than dinucleotides in the genomic DNA of vertebrates^6,50^. The mononucleotides detected have an abundance similar to that of the trinucleotides (around 16%) and the most abundant motifs are A and T, which account for about 81%. In primates, mononucleotides are represented by A and T motifs and are the most frequent among SSRs^6^. In relation to the distribution of SSRs throughout the genome, using mapped BAC sequencing as sampling, there is some variation among the particular chromosomes, and it is noteworthy that chromosome 1 is one of those with the lowest SSR abundance values.

Using SSR probes in FISH experiments, we observed that some di-and tetra-nucleotide microsatellites produce the strongest FISH signals. The bioinformatic analysis of the BAC clones indicated that the AC motif has the highest relative abundance value. Our SSR-FISH results support this datum: AC shows up in clusters with the brightest and most intense signal in almost all chromosomes. Furthermore, the GACA, GATA and AG elements showed similar patterns after applying the FISH technique to localize them. Hence, these four microsatellites are probably present as an established combination of repetitive elements in the heterochromatin of sole. Mononucleotide probes (A) and (C) were found scattered throughout the chromosomes. Using FISH there was no clear correspondence between the frequency of the microsatellite motif and the intensity of the signal, since in our study the AAT motif with frequency of 28% gave no signal while the C motifs with 18% and AG with 14% gave more intense signals. In fish species such as *D. rerio, Rineloricaria latirostris and Steindachneridion scripta*, these repetitive sequences tend to be grouped in the telomeric and centromeric regions^59^.

In relation to repeated elements, we have identified a total of 4686, from which, 4121 were TE elements. These TE elements showed a total length of 467144 bp (5.94% of the genome analyzed). When compared with other fish, this proportion is similar to that found in the two smallest reported genomes of teleost fish, the green spotted pufferfish, and the fugu (*T. rubripes*), with genome sizes of approximately 342 and 393 Mb, respectively, that contain only ~ 6% of their DNA derived from TE^60^. The proportion of TEs in stickleback, cod and European eel, with values of 12–15% of their genome, is twice that observed in the *S. senegalensis* genome^13^. In the group of tilapia, platyfish, medaka and spotted gar the proportion of TEs is even higher, with values between 20 and 30%; and the proportion observed in the coelacanth is 25% of the genome^13^. The number and proportions of TEs differ widely among genomes of actinopterygian (ray-finned) fishes, especially teleosts. In fact, a large part of the zebrafish genome (~1.4 Gb) consists of TEs (55%)^14^. The abundance of TEs seems to be the main determining factor of genome size in this group^13,61^. However, TEs proportion in the small genome of tetraodon (representing just 7.13% of its genome) and in other vertebrates as birds (TE content values ranging 8–10%) are also close to those found in sole^13,61^.

Considering the genome distribution of TEs by chromosome, our results show a heterogeneous distribution. Class II TEs (DNA transposon) cover almost 55% of total Repetitive DNA found in the *S. senegalensis* BAC sequences analized and it is similar to the 60% of Class II TEs detected in cichlids and somewhat greater than the 39% of the same type of TEs in the zebrafish genome. In *S. senegalensis* the retrotransposons (Class I) account for 33%, with a coverage of LINES of 15%, the most abundant with 717 matches, coverage of SINES (short interspersed elements) of 10%, and of LTRs of almost 8%, whereas in cichlids and zebrafish retrotransposons represent less than 12% of each type^14,62^. The DNA transposons (Class II) were the most abundant with 55% of the TEs found, these being the class with the highest presence (900 elements and abundance of 19%) in the *S. senegalensis* genome analyzed. In particular, two main TIR (Translocated Intimin Receptor) families (hAT and Tc-Mariner), with many subfamilies, constitute the largest fraction of DNA transposons in the sole genome. To a lesser extent, Harbinger has also been detected.

The TIR family of hAT transposons is worth mentioning, given its coverage of 1% of the genome studied. This value represents a coverage ten times higher than that detected in the coelacanth (0.11%) and Lung fish (0.1%)^13^. The hAT transposons are also found in the genomes of mammals, including humans, where they are the most abundant DNA transposons and comprise 1.55% (195 Mb) of the total genome^22^. In chicken, values similar to those of fish have been found (0.1%) and the value detected in salamander (0.63%) is also lower than that of sole^13^. The highest value of genome coverage in the hAT superfamily: 6.10% was detected in frog^1^. Few data are available on the role played by the hAT superfamily in fish; however, it is known that none of the hAT elements in the human genome have been active during the last 50 million years^22^. In vertebrates, most hAT transposons are inactive, since host cells have developed the mechanism of vertical inactivation to silence and prevent the deleterious effects of active transposons on genome stability^63^.

We have found a sequence that presents homology with the Rex retrotransposon of many species. The abundance of TEs of the Rex type detected mostly in the chromosome pair 1 of the *S. senegalensis* genome, raises the hypothesis that this chromosome could be a proto-sex chromosome^29^. It is known that Rex-type transposons are very important in the evolution of the eukaryotic genome, and participate in processes of chromosomal rearrangement^64^ and chromosomal sex differentiation^65–67^, which are involved in sexual differentiation. Several authors have also associated these transposable and retro-transposable elements with chromosomal sex differentiation in groups of fish such as Cyprinodontiformes^68^, Characiformes^69^, and Beloniformes^70^. Indeed, in the Cyprinodontiforme *Semaprochilodus taeniurus*, Terencio *et al*.^69^ observed a significant increase in the size of the W chromosome due to repetitive DNA accumulation, and among these DNA sequences was *Rex*1.

In *O. nitolicus*, Rex elements are concentrated in the first pair of chromosomes^18^. In this species, the first pair of chromosomes seems to correspond to the sex chromosomes^71^, possibly originated from fusion processes^72^. The location of the Rex1 elements in the chromosome pair 1 could have had some role in chromosomal rearrangements of the *S. senegalensis* genome, as occurs in *O. nitolicus*^18^.

In *S. senegalensis*, our SSR-FISH results showed a higher concentration in subtelomeric positions of several probes that are probably present as a combination of repetitive elements in the heterochromatin of sole. This heterochromatin is present in metacentric chromosomes, such as chromosome pair 1. In addition, one of the BACs where Rex1 presented the highest length and abundance values (BAC10-L10), was found in a subtelomeric position in chromosome 1. Hence, this subtelomeric region could be comprised of heterochromatin in which (or adjacent to which) the Rex1 retrotransposon could occur.

It has been described that the preferential position of Rex1, Rex2 and Rex6 genes in heterochromatic regions of the genomes of some fish^73,74^ could indicate some mechanism of regulation of these elements that impedes or prevents excessive dispersion and propagation in the genome, since the presence of heterochromatin could be regulating, through epigenetic mechanisms, the dispersion of these sequences without modifying their sequence^75^. Several studies have shown a relationship between the preferential presence of repetitive sequences in sexual chromosomes and heterochromatin regions. Thus, in *Harttia carvalhoi* (Loricariidae) it has been discussed how the location of the retroelements Rex1, Rex3 and Rex6 in the pericentromeric region of an X chromosome could have influenced its fission, which led to the formation of chromosomes Y1 and Y2^69^.

The first reference to the existence of Rex1 was published by Volff *et al*.^68^, after finding an insert in a cosmid from the Y sex chromosome of *X. maculatus* that revealed a sequence encoding a product with similarities to the RT of non-LTR retrotransposons. That sequence was called Rex1-XimJ^68^. After a wide analysis, the phylogeny of Rex1 sequences was explained by the presence of four major ancient lineages in fish genomes. The lineage 4 contained sequences from *O. latipes* and *O. niloticus* among others. Lineage 4 is observed in all Acanthopterygii, but not in *C. carpio*, *D. rerio* or *O. mykiss*, among others^68^. In the Rex1 phylogenetic tree constructed, the Senegalese sole Rex1 retrotransposon could be associated with one of the four major ancient lineages in fish genomes, in which it is included *O. latipes*.

One of the hypotheses to explain the wide distribution of the lineage 4 of Rex1in fishes is the possibility of horizontal transfer^68^. Horizontal transfer has been well documented for some DNA transposons and for LTR retrotransposons^76^. The possibility of a horizontal transfer (HT) event between phylogenetically distant species (Perciformes and Batrachoidiformes orders) has been recently reported in fishes^77^. It has also been demonstrated that 5 S rRNA genes and retro-transposons can interact with one another^78^, and this interaction might be the cause of the pattern of evolution and the dispersed arrangement of some organisms. Therefore a putative role of the Rex1 retrotransposon, and its presence in a heterochromatic region of *S. senegalensis*, in the evolution of this putative sex proto-chromosome 1 should be not rejected. On the other hand, the chromosome pair 15 has also shown high abundance of Rex1 sequences in the BACs localized in this pair. In a previous work, the BAC 19-J21 also localized in this chromosome, and it carried the SOX9 gene^29^. In the Prochilodontidae fish family, the W chromosome of *Semaprochilodus taeniurus* species, has significantly increased in size due to the accumulation of repetitive DNAs, like the Rex1 retro-element, with the consequent differentiation of the ZZ/ZW system of sex chromosomes^69^. In that study, one of the W-specific fragments showed high similarity with the transcription factor of the SOX9 gene in *T. rubripes*. The SOX9 is a gene related to sex determination in many organisms and is present in the BAC 19-J21 in *S. senegalensis*. Hence the presence of the Rex1 gene in regions where it occurs, and the role it has played in certain events related to sex determination, must be taken into account in studies of the evolution of the Senegalese sole genome.

## Conclusions

Our work represents a first approach to the study of the repetitive elements of the genome of the Senegalese sole (*S. senegalensis*). The analysis of the location of SSR allowed the description of large clusters of microsatellites in centromeric and subtelomeric positions, as well as the study of their composition by bioinformatic analysis. These results reflect a prevalence of A-rich repetitions during the evolution of this species as occurs in the genomes of other teleostats. The study of TEs revealed that the most abundant family in the genome of this flatfish is the hAT, as well as the discovery of a transposable Class I element, Rex, in the largest metacentric chromosome pair, recently described as a possible proto-sex chromosome. The presence of this element on this chromosome and its position in a heterochromatin region might have been relevant during the evolution of the chromosome. Our results present an important advance on the evolution of the *S. senegalensis* genome through the analysis of the distribution and quantification of repetitive elements and the role that Rex 1 may have played in certain events related to sex determination.

## Supplementary information

Supplementary Dataset 1

Supplementary Dataset 2

Supplementary Dataset 3
