# Supplementary material for: Integrative genetic map of repetitive DNA in the sole Solea senegalensis genome shows a Rex transposon located in a proto-sex chromosome
Source: Sci Rep. 2019 Nov 20;9:17146. doi: 10.1038/s41598-019-53673-6 (PMC6868151; doi:10.1038/s41598-019-53673-6)
Supplement: Supplementary file 1 — Supplementary Dataset 1 [file 41598_2019_53673_MOESM1_ESM.docx]

**Integrative genetic map of repetitive DNA in the sole *Solea senegalensis* genome shows a Rex transposon located in a proto-sex chromosome**

**Emilio García*^1^, Ismael Cross*^1^, Silvia Portela-Bens^1^, María E. Rodríguez^1^, Aglaya García-Angulo^1^, Belén Molina^1^, Angeles Cuadrado^2^, Thomas Liehr^3^, Laureana Rebordinos^1^**

**Supplementary File 1**

Summary of BAC clones analysed in the present work, displaying number of contigs per BACs, BAC sizes and their chromosome location in *S. senegalensis* genome after using mBAC-FISH technique.

| Chromosome | BAC Id. | BAC size | No. Contigs | Reference |
| --- | --- | --- | --- | --- |
| 1 | 10-L10 | 65053 | 3 | Garcia-Angulo et al (2018) |
| 1 | 16-E16 | 43095 | 3 | Garcia-Angulo et al (2018) |
| 1 | 5-K5 | 591556 | 351 | Merlo et al (2017) |
| 1 | 48-P7 | 242510 | 7 | Garcia-Angulo et al (2018) |
| 1 | 48-K7 | 179229 | 4 | Portela-Bens et al (2017) |
| 1 | 56-H24 | 155006 | 18 | Garcia-Angulo et al (2018) |
| 1 | 12-D22 | 56534 | 27 | Merlo et al (2017) |
| 1 | 36-D3 | 65243 | 17 | Garcia-Angulo et al (2018) |
| 1 | 1-C2 | 44432 | 1 | Garcia-Angulo et al (2018) |
| 1 | 52-C17 | 185039 | 5 | Garcia-Angulo et al (2018) |
| 1 | 13-G1 | 15554 | 3 | Garcia-Angulo et al (2018) |
| 2 | 4-D15 | 75166 | 11 | This work |
| 2 | 6-P22 | 53266 | 43 | Portela-Bens et al (2017) |
| 2 | 19-J21 | 56544 | 17 | Portela-Bens et al (2017) |
| 2 | 52-G10 | 171874 | 8 | This work |
| 2 | 46-C5 | 190621 | 5 | This work |
| 2 | 36-I3 | 34866 | 8 | This work |
| 2 | 21-O23 | 53932 | 5 | Portela-Bens et al (2017) |
| 2 | 36-K1 | 30968 | 11 | This work |
| 3 | 9-J4 | 140126 | 3 | This work |
| 4 | 46-B2 | 175688 | 6 | This work |
| 4 | 12-N15 | 162898 | 17 | Portela-Bens et al (2017) |
| 4 | 30-J4 | 372766 | 36 | Merlo et al (2017) |
| 4 | 8-A23 | 100967 | 4 | This work |
| 4 | 36-J2 | 56196 | 5 | This work |
| 4 | 3-C15 | 78966 | 10 | This work |
| 4 | 36-H3 | 65179 | 18 | This work |
| 4 | 12-D24 | 183408 | 5 | This work |
| 4 | 36-H2 | 75632 | 32 | This work |
| 6 | 48-K7 | 179229 | 4 | Portela-Bens et al (2017) |
| 6 | 16-E16 | 43095 | 3 | Garcia-Angulo et al (2018) |
| 6 | 10-K23 | 96482 | 23 | Portela-Bens et al (2017) |
| 6 | 20-D18 | 48004 | 38 | Portela-Bens et al (2017) |
| 7 | 7-H22 | 120282 | 57 | Garcia-Cegarra et al (2013) |
| 7 | 19-H19 | 70801 | 5 | Portela-Bens et al (2017) |
| 7 | 8-O7 | 175561 | 11 | Portela-Bens et al (2017) |
| 8 | 31-A1 | 76388 | 9 | This work |
| 8 | 31-A2 | 70097 | 7 | This work |
| 9 | 32-B8 | 196621 | 14 | Portela-Bens et al (2017) |
| 10 | 15-I19 | 203915 | 8 | This work |
| 11 | 4-E10 | 75452 | 21 | This work |
| 11 | 31-F1 | 53125 | 14 | This work |
| 12 | 30-J4 | 372766 | 36 | Merlo et al (2017) |
| 12 | 35-D17 | 111328 | 4 | This work |
| 12 | 13-F2 | 26326 | 23 | This work |
| 13 | 29-D4 | 198050 | 6 | This work |
| 13 | 8-O7 | 175561 | 11 | Portela-Bens et al (2017) |
| 14 | 21-O23 | 53932 | 5 | Portela-Bens et al (2017) |
| 15 | 19-J21 | 56544 | 17 | Portela-Bens et al (2017) |
| 15 | 4-F12 | 68549 | 16 | This work |
| 15 | 16-E16 | 43095 | 3 | Portela-Bens et al (2017) |
| 15 | 36-E3 | 43011 | 16 | This work |
| 16 | 30-P17 | 84700 | 2 | This work |
| 16 | 52-E7 | 182264 | 3 | This work |
| 16 | 9-N8 | 50351 | 4 | Garcia-Cegarra et al (2013) |
| 17 | 31-N1 | 69340 | 21 | This work |
| 17 | 63-A7 | 184023 | 7 | This work |
| 18 | 36-M2 | 81656 | 15 | This work |
| 19 | 12-K6 | 19946 | 3 | Garcia-Cegarra et al (2013) |
| 19 | 13-F4 | 88689 | 3 | This work |
| 19 | 31-C1 | 169459 | 28 | This work |
| 20 | 4-M14 | 291331 | 171 | This work |
| 21 | 63-A3 | 248683 | 145 | This work |
| 21 | 30-H22 | 144595 | 68 | Portela-Bens et al (2017) |
