# Supplementary material for: Integrative genetic map of repetitive DNA in the sole Solea senegalensis genome shows a Rex transposon located in a proto-sex chromosome
Source: Sci Rep. 2019 Nov 20;9:17146. doi: 10.1038/s41598-019-53673-6 (PMC6868151; doi:10.1038/s41598-019-53673-6)
Supplement: Supplementary file 2 — Supplementary Dataset 2 [file 41598_2019_53673_MOESM2_ESM.docx]

**Integrative genetic map of repetitive DNA in the sole *Solea senegalensis* genome shows a Rex transposon located in a proto-sex chromosome**

**Emilio García*^1^, Ismael Cross*^1^, Silvia Portela-Bens^1^, María E. Rodríguez^1^, Aglaya García-Angulo^1^, Belén Molina^1^, Angeles Cuadrado^2^, Thomas Liehr^3^, Laureana Rebordinos^1^**

**Supplementary file 2**

Summary of statistics of the *S. senegalensis* SSR analysis: Abundance and coverage of SSR per chromosome measured from BAC sequences analysis. NL= number of loci.

| Chromosome | Length | N SSR (NL) | Coverage | NL abundance (N/1Mb) | Coverage abundance (Cov/1Mb) |
| --- | --- | --- | --- | --- | --- |
| Chr. 1 | 1643251 | 622 | 15849 | 378,52 | 9644,91 |
| Chr. 2 | 667237 | 515 | 15614 | 771,84 | 23400,98 |
| Chr. 3 | 140126 | 75 | 1923 | 535,23 | 13723,36 |
| Chr. 4 | 1271700 | 765 | 24411 | 601,56 | 19195,56 |
| Chr. 6 | 366810 | 226 | 6172 | 616,12 | 16826,15 |
| Chr. 7 | 366644 | 149 | 4196 | 406,39 | 11444,34 |
| Chr. 8 | 146485 | 94 | 2550 | 641,70 | 17407,93 |
| Chr. 9 | 196621 | 135 | 3493 | 686,60 | 17765,14 |
| Chr. 10 | 203915 | 137 | 3092 | 671,85 | 15163,18 |
| Chr. 11 | 128577 | 74 | 1495 | 575,53 | 11627,27 |
| Chr. 12 | 510420 | 353 | 10715 | 691,59 | 20992,52 |
| Chr. 13 | 373611 | 182 | 5055 | 487,14 | 13530,12 |
| Chr. 14 | 53932 | 37 | 852 | 686,05 | 15797,67 |
| Chr. 15 | 211199 | 160 | 4832 | 757,58 | 22878,90 |
| Chr. 16 | 317315 | 148 | 3869 | 466,41 | 12192,93 |
| Chr. 17 | 253363 | 237 | 10591 | 935,42 | 41801,68 |
| Chr. 18 | 81656 | 69 | 1630 | 845,01 | 19961,79 |
| Chr. 19 | 278094 | 141 | 4793 | 507,02 | 17235,18 |
| Chr. 20 | 291331 | 67 | 1590 | 229,98 | 5457,71 |
| Chr. 21 | 393278 | 162 | 4766 | 411,92 | 12118,65 |
