# Supplementary material for: Integrative genetic map of repetitive DNA in the sole Solea senegalensis genome shows a Rex transposon located in a proto-sex chromosome
Source: Sci Rep. 2019 Nov 20;9:17146. doi: 10.1038/s41598-019-53673-6 (PMC6868151; doi:10.1038/s41598-019-53673-6)
Supplement: Supplementary file 3 — Supplementary Dataset 3 [file 41598_2019_53673_MOESM3_ESM.docx]

**Integrative genetic map of repetitive DNA in the sole *Solea senegalensis* genome shows a Rex transposon located in a proto-sex chromosome**

**Emilio García*^1^, Ismael Cross*^1^, Silvia Portela-Bens^1^, María E. Rodríguez^1^, Aglaya García-Angulo^1^, Belén Molina^1^, Angeles Cuadrado^2^, Thomas Liehr^3^, Laureana Rebordinos^1^**

**Supplementary File 3**

Species, databases and labels of the sequences used to make the Rex-1 phylogenetic tree.

| #ID | Label | Dabase | Species | Common name |
| --- | --- | --- | --- | --- |
| REX1-1_AFC | Cichlidae_1 | Repbase | *Cichlidae* | African cichlid |
| REX1-2_AFC | Cichlidae_2 | Repbase | *Cichlidae* | African cichlid |
| REX1-3_AFC | Cichlidae_3 | Repbase | *Cichlidae* | African cichlid |
| REX1-4_AFC | Cichlidae_4 | Repbase | *Cichlidae* | African cichlid |
| Rex1-5_AFC | Cichlidae_5 | Repbase | *Cichlidae* | African cichlid |
| AM1_scaffold:AstMex102:KB882165.1:2163518:2164831:-1 | A.mexicanus_1 | Ensemble | *Astianax mexicanus* | Cave fish |
| AM2_scaffold:AstMex102:KB882247.1:1048916:1050222:1 | A.mexicanus_2 | Ensemble | *Astianax mexicanus* | Cave fish |
| AM3_scaffold:AstMex102:KB882294.1:288834:290080:1 | A.mexicanus_3 | Ensemble | *Astianax mexicanus* | Cave fish |
| AM4_scaffold:AstMex102:KB882162.1:1882982:1884169:1 | A.mexicanus_4 | Ensemble | *Astianax mexicanus* | Cave fish |
| AM5_scaffold:AstMex102:KB882197.1:907654:908779:1 | A.mexicanus_5 | Ensemble | *Astianax mexicanus* | Cave fish |
| DR1_scaffold:GRCz10:KN149952.1:53884:56420:1 | D.rerio_1 | Ensemble | *Danio rerio* | Zebrafish |
| DR2_chromosome:GRCz10:23:42573128:42575670:-1 | D.rerio_2 | Ensemble | *Danio rerio* | Zebrafish |
| DR3_chromosome:GRCz10:14:15144268:15146786:1 | D.rerio_3 | Ensemble | *Danio rerio* | Zebrafish |
| DR4_chromosome:GRCz10:8:46473559:46476057:1 | D.rerio_4 | Ensemble | *Danio rerio* | Zebrafish |
| DR5_chromosome:GRCz10:21:11242564:11245060:1 | D.rerio_5 | Ensemble | *Danio rerio* | Zebrafish |
| Rex1-15_DRe | D.rerio_6 | Repbase | *Danio rerio* | Zebrafish |
| Rex1-19_DRe | D.rerio_7 | Repbase | *Danio rerio* | Zebrafish |
| REX1-2_DR | D.rerio_8 | Repbase | *Danio rerio* | Zebrafish |
| Rex1-20_DRe | D.rerio_9 | Repbase | *Danio rerio* | Zebrafish |
| Rex1-21_DRe | D.rerio_10 | Repbase | *Danio rerio* | Zebrafish |
| Rex1-23_DRe | D.rerio_11 | Repbase | *Danio rerio* | Zebrafish |
| Rex1-26_DRe | D.rerio_12 | Repbase | *Danio rerio* | Zebrafish |
| Rex1-28_Dre | D.rerio_13 | Repbase | *Danio rerio* | Zebrafish |
| Rex1-29_DRe | D.rerio_14 | Repbase | *Danio rerio* | Zebrafish |
| REX1-3_DR | D.rerio_15 | Repbase | *Danio rerio* | Zebrafish |
| Rex1-30_DRe | D.rerio_16 | Repbase | *Danio rerio* | Zebrafish |
| Rex1-31_DRe | D.rerio_17 | Repbase | *Danio rerio* | Zebrafish |
| Rex1-38_DRe | D.rerio_18 | Repbase | *Danio rerio* | Zebrafish |
| REX1-4_DR | D.rerio_19 | Repbase | *Danio rerio* | Zebrafish |
| Rex1-40_DRe | D.rerio_20 | Repbase | *Danio rerio* | Zebrafish |
| Rex1-40B3_DR | D.rerio_21 | Repbase | *Danio rerio* | Zebrafish |
| Rex1-43_DRe | D.rerio_22 | Repbase | *Danio rerio* | Zebrafish |
| REX1-5_DR | D.rerio_23 | Repbase | *Danio rerio* | Zebrafish |
| Rex1-50_DR | D.rerio_24 | Repbase | *Danio rerio* | Zebrafish |
| Rex1-53_DR | D.rerio_25 | Repbase | *Danio rerio* | Zebrafish |
| Rex1-54_DR | D.rerio_26 | Repbase | *Danio rerio* | Zebrafish |
| Rex1-55_DR | D.rerio_27 | Repbase | *Danio rerio* | Zebrafish |
| Rex1-56_DR | D.rerio_28 | Repbase | *Danio rerio* | Zebrafish |
| Rex1-57_DR | D.rerio_29 | Repbase | *Danio rerio* | Zebrafish |
| Rex1-58_DR | D.rerio_30 | Repbase | *Danio rerio* | Zebrafish |
| Rex1-59_DR | D.rerio_31 | Repbase | *Danio rerio* | Zebrafish |
| Rex1-6_DR | D.rerio_32 | Repbase | *Danio rerio* | Zebrafish |
| Rex1-60_DR | D.rerio_33 | Repbase | *Danio rerio* | Zebrafish |
| Rex1-61_DR | D.rerio_34 | Repbase | *Danio rerio* | Zebrafish |
| REX1-7_DR | D.rerio_35 | Repbase | *Danio rerio* | Zebrafish |
| REX1-8_DR | D.rerio_36 | Repbase | *Danio rerio* | Zebrafish |
| REX1-9_DR | D.rerio_37 | Repbase | *Danio rerio* | Zebrafish |
| REX1-1_DR | D.rerio_38 | Repbase | *Danio rerio* | Zebrafish |
| Rex1-1_EL | E.lucius_1 | Repbase | *Esox lucius* | Northern pike |
| Rex1-1B_EL | E.lucius_2 | Repbase | *Esox lucius* | Northern pike |
| Rex1-3_EL | E.lucius_3 | Repbase | *Esox lucius* | Northern pike |
| Rex1-4_EL | E.lucius_4 | Repbase | *Esox lucius* | Northern pike |
| Rex1-5_EL | E.lucius_5 | Repbase | *Esox lucius* | Northern pike |
| REX1_FS | Fundulus sp. | Repbase | *Fundulus sp. 'Laguna de Labradores'* | Fundulus |
| GA1_group:BROADS1:groupXIX:5684077:5686043:-1 | G.aculeatus_1 | Ensemble | *Gasterosteus aculeatus* | Stickleback |
| GA2_group:BROADS1:groupVII:11379802:11381439:1 | G.aculeatus_2 | Ensemble | *Gasterosteus aculeatus* | Stickleback |
| GA3_scaffold:BROADS1:scaffold_27:4360099:4361401:-1 | G.aculeatus_3 | Ensemble | *Gasterosteus aculeatus* | Stickleback |
| GA4_group:BROADS1:groupIV:17241161:17242470:-1 | G.aculeatus_4 | Ensemble | *Gasterosteus aculeatus* | Stickleback |
| GA5_scaffold:BROADS1:scaffold_80:480561:481751:-1 | G.aculeatus_5 | Ensemble | *Gasterosteus aculeatus* | Stickleback |
| REX1-1_GA | G.aculeatus_6 | Repbase | *Gasterosteus aculeatus* | Stickleback |
| Rex1-2_GA | G.aculeatus_7 | Repbase | *Gasterosteus aculeatus* | Stickleback |
| Rex1-3_GA | G.aculeatus_8 | Repbase | *Gasterosteus aculeatus* | Stickleback |
| GM3_contig::contig285937:33:2300:-1 | Gadus morua | Ensemble | *Gadus morua* | Cod |
| LO1_chromosome:LepOcu1:LG12:27307519:27310028:-1 | L.oculatus_1 | Ensemble | *Lepisosteus oculatus* | Spotted gar |
| LO2_scaffold:LepOcu1:JH591410.1:625708:628209:1 | L.oculatus_2 | Ensemble | *Lepisosteus oculatus* | Spotted gar |
| LO3_chromosome:LepOcu1:LG7:34681474:34683968:-1 | L.oculatus_3 | Ensemble | *Lepisosteus oculatus* | Spotted gar |
| LO4_chromosome:LepOcu1:LG7:24563161:24565646:1 | L.oculatus_4 | Ensemble | *Lepisosteus oculatus* | Spotted gar |
| LO5_chromosome:LepOcu1:LG5:25775845:25778323:1 | L.oculatus_5 | Ensemble Ensemble | *Lepisosteus oculatus* | Spotted gar |
| OL1_scaffold:MEDAKA1:scaffold703:83644:86197:-1 | O.latipes_1 | Ensemble | *Oryzias latipes* | Medaka |
| OL2_ultracontig:MEDAKA1:ultracontig147:244827:247354:-1 | O.latipes_2 | Ensemble | *Oryzias latipes* | Medaka |
| OL3_chromosome:MEDAKA1:17:376791:379261:-1 | O.latipes_3 | Ensemble | *Oryzias latipes* | Medaka |
| OL4_scaffold:MEDAKA1:scaffold683:35002:36979:-1 | O.latipes_4 | Ensemble | *Oryzias latipes* | Medaka |
| OL5_chromosome:MEDAKA1:3:5628283:5630180:-1 | O.latipes_5 | Ensemble | *Oryzias latipes* | Medaka |
| Rex1-1_OL | O.latipes_6 | Repbase | *Oryzias latipes* | Medaka |
| Rex1-2_OL | O.latipes_7 | Repbase | *Oryzias latipes* | Medaka |
| Rex1-3_OL | O.latipes_8 | Repbase | *Oryzias latipes* | Medaka |
| Rex1-4_OL | O.latipes_9 | Repbase | *Oryzias latipes* | Medaka |
| Rex1-5_OL | O.latipes_10 | Repbase | *Oryzias latipes* | Medaka |
| Rex1-6_OL | O.latipes_11 | Repbase | *Oryzias latipes* | Medaka |
| ON1_scaffold:Orenil1.0:GL831180.1:651167:653729:1 | O.niloticus_1 | Ensemble | *Oreochromis niloticus* | Tilapia |
| ON2_scaffold:Orenil1.0:GL831276.1:1961235:1963778:1 | O.niloticus_2 | Ensemble | *Oreochromis niloticus* | Tilapia |
| ON3_scaffold:Orenil1.0:GL831515.1:317604:320128:1 | O.niloticus_3 | Ensemble | *Oreochromis niloticus* | Tilapia |
| ON5_scaffold:Orenil1.0:GL832145.1:13230:15626:-1 | O.niloticus_4 | Ensemble | *Oreochromis niloticus* | Tilapia |
| PF1_scaffold:PoeFor_5.1.2:KI520726.1:13469:15744:-1 | P.formosa_1 | Ensemble | *Poecillia formosa* | Amazon molly |
| PF2_scaffold:PoeFor_5.1.2:KI520035.1:165716:167248:1 | P.formosa_2 | Ensemble | *Poecillia formosa* | Amazon molly |
| PF3_scaffold:PoeFor_5.1.2:KI520211.1:177172:178603:-1 | P.formosa_3 | Ensemble | *Poecillia formosa* | Amazon molly |
| PF4_scaffold:PoeFor_5.1.2:KI519742.1:522116:523528:1 | P.formosa_4 | Ensemble | *Poecillia formosa* | Amazon molly |
| Rex1-1_PM | P.marinus_1 | Repbase | *Petromyzon marinus* | Lamprey |
| Rex1-2_PM | P.marinus_2 | Repbase | *Petromyzon marinus* | Lamprey |
| REX1_CY | Poeciliidae | Repbase | *Poeciliidae* | Poecilid |
| Rex1-1_SSa | S.salar_1 | Repbase | *Salmo salar* | Salmon |
| Rex1-10_SSa | S.salar_2 | Repbase | *Salmo salar* | Salmon |
| Rex1-11_SSa | S.salar_3 | Repbase | *Salmo salar* | Salmon |
| Rex1-2_SSa | S.salar_4 | Repbase | *Salmo salar* | Salmon |
| Rex1-3_SSa | S.salar_5 | Repbase | *Salmo salar* | Salmon |
| Rex1-3B_SSa | S.salar_6 | Repbase | *Salmo salar* | Salmon |
| Rex1-3C_SSa | S.salar_7 | Repbase | *Salmo salar* | Salmon |
| Rex1-3D_SSa | S.salar_8 | Repbase | *Salmo salar* | Salmon |
| Rex1-3E_SSa | S.salar_9 | Repbase | *Salmo salar* | Salmon |
| Rex1-4_SSa | S.salar_10 | Repbase | *Salmo salar* | Salmon |
| Rex1-5_SSa | S.salar_11 | Repbase | *Salmo salar* | Salmon |
| Rex1-6_SSa | S.salar_12 | Repbase | *Salmo salar* | Salmon |
| Rex1-7_SSa | S.salar_13 | Repbase | *Salmo salar* | Salmon |
| Rex1-8_SSa | S.salar_14 | Repbase | *Salmo salar* | Salmon |
| Rex1-9_SSa | S.salar_15 | Repbase | *Salmo salar* | Salmon |
| Chr_1_BAC_10_L10_contig00002:9850-12400 Rex1-1_PM | S.senegalensis | BAC | *Solea senegalensis* | Senegalese sole |
| TN1_chromosome:TETRAODON8:Un_random:55787774:55790286:1 | T.nigroviridis_1 | Ensemble | *Tetraodon nigroviridis* | Tetraodon |
| TN2_chromosome:TETRAODON8:Un_random:51662343:51664838:-1 | T.nigroviridis_2 | Ensemble | *Tetraodon nigroviridis* | Tetraodon |
| TN3_chromosome:TETRAODON8:Un_random:56050744:56053235:1 | T.nigroviridis_3 | Ensemble | *Tetraodon nigroviridis* | Tetraodon |
| TN4_chromosome:TETRAODON8:Un_random:55026367:55028848:-1 | T.nigroviridis_4 | Ensemble | *Tetraodon nigroviridis* | Tetraodon |
| TN5_chromosome:TETRAODON8:Un_random:6416588:6418835:1 | T.nigroviridis_5 | Ensemble | *Tetraodon nigroviridis* | Tetraodon |
| TR1_scaffold:FUGU4:scaffold_286:39301:41558:-1 | T.rubripes_1 | Ensemble | *Takifugu rubripes* | Fugu |
| TR2_scaffold:FUGU4:scaffold_3727:4906:7163:-1 | T.rubripes_2 | Ensemble | *Takifugu rubripes* | Fugu |
| TR3_scaffold:FUGU4:scaffold_47:270297:272028:-1 | T.rubripes_3 | Ensemble | *Takifugu rubripes* | Fugu |
| TR4_scaffold:FUGU4:scaffold_300:95495:97209:-1 | T.rubripes_4 | Ensemble | *Takifugu rubripes* | Fugu |
| TR5_scaffold:FUGU4:scaffold_161:297292:298993:1 | T.rubripes_5 | Ensemble | *Takifugu rubripes* | Fugu |
| Rex1-1_FR | T.rubripes_6 | Repbase | *Takifugu rubripes* | Fugu |
| Rex1-1B_FR | T.rubripes_7 | Repbase | *Takifugu rubripes* | Fugu |
| Rex1-2_FR | T.rubripes_8 | Repbase | *Takifugu rubripes* | Fugu |
| Rex1-3_FR | T.rubripes_9 | Repbase | *Takifugu rubripes* | Fugu |
| XM1_scaffold:Xipmac4.4.2:JH558418.1:25442:26996:1 | X.maculatus_1 | Ensemble | *Xiphophorus maculatus* | Platyfish |
| XM2_scaffold:Xipmac4.4.2:JH558658.1:8860:10264:-1 | X.maculatus_2 | Ensemble | *Xiphophorus maculatus* | Platyfish |
| XM3_scaffold:Xipmac4.4.2:JH557238.1:45054:46311:-1 | X.maculatus_3 | Ensemble | *Xiphophorus maculatus* | Platyfish |
| XM4_scaffold:Xipmac4.4.2:AGAJ01054897.1:1:1260:1 | X.maculatus_4 | Ensemble | *Xiphophorus maculatus* | Platyfish |
| XM5_scaffold:Xipmac4.4.2:JH556859.1:610664:611919:-1 | X.maculatus_5 | Ensemble | *Xiphophorus maculatus* | Platyfish |
